# Combination of Geriatric Nutritional Risk Index and Carcinoembryonic Antigen to Predict the Survival of Patients With Colorectal Cancer

**DOI:** 10.3389/fnut.2022.902080

**Published:** 2022-06-30

**Authors:** Hailun Xie, Lishuang Wei, Guanghui Yuan, Mingxiang Liu, Yanren Liang, Shunhui Gao, Qiwen Wang, Xin Lin, Shuangyi Tang, Jialiang Gan

**Affiliations:** ^1^Department of Colorectal and Anal Surgery, The First Affiliated Hospital, Guangxi Medical University, Nanning, China; ^2^Guangxi Key Laboratory of Enhanced Recovery After Surgery for Gastrointestinal Cancer, Nanning, China; ^3^Department of Geriatric Respiratory Disease Ward, The First Affiliated Hospital, Guangxi Medical University, Nanning, China; ^4^Grade 2018, Department of Clinical Medicine, Guangxi Medical University, Nanning, China; ^5^Department of Pharmacy, The First Affiliated Hospital, Guangxi Medical University, Nanning, China

**Keywords:** GNRI, CEA, colorectal cancer, prognosis, surgery

## Abstract

**Background:**

This study explored the value of the combination of Geriatric Nutritional Risk Index (GNRI) and carcinoembryonic antigen (CEA) for the prognosis assessment of CRC patients.

**Methods:**

This study retrospectively enrolled 1,014 CRC patients who underwent surgery between 2012 and 2014. Kaplan-Meier and log-rank tests were used to compare survival differences. Cox proportional hazards regression analysis was used to assess risk factors associated with progression-free survival (PFS) and overall survival (OS). Nomograms were constructed to predict the prognosis of CRC patients. Randomized internal validation was used to confirm the predictive accuracy of the prognostic nomograms.

**Results:**

The GNRI-CEA score was established by combining GNRI and CEA. Compared with patients with normal GNRI-CEA scores, patients with mild/moderate/severe GNRI-CEA scores had significantly lower survival (PFS, 68.99% vs. 57.75% vs. 41.34% vs. 31.36%, *p* < 0.001; OS, 68.99% vs. 57.75% vs. 41.34% vs. 31.36%, *p* < 0.001). The GNRI-CEA score is an independent factor predicting the prognosis of CRC patients. The risk of death was twofold higher in patients with low GNRI and high CEA than in those with both normal GNRI and CEA [PFS, hazard ratio (HR), 2.339; 95% confidence interval (CI), 1.656–3.303; *p* < 0.001; OS, HR, 2.340; 95% CI, 1.645–3.329; *p* < 0.001]. Prognostic nomograms had good resolution and accuracy in predicting 1–5 year PFS and OS. Randomized internal validation showed that the nomograms were reliable.

**Conclusion:**

The combination of GNRI and CEA can effectively stratify the prognosis of CRC patients. The nomogram established based on the two indices can provide a personalized reference for prognostic assessment and clinical decision-making for CRC patients.

## Introduction

Colorectal cancer (CRC) is a malignancy with a high mortality rate that affects human health and longevity. CRC is the third most common cancer and the second leading cause of cancer-related death worldwide (1). In 2019, there were an estimated 2.17 million new cases of CRC and 1.09 million CRC-related deaths worldwide. CRC directly causes 24.3 million disability-adjusted life years, second only to tracheal, bronchus, and lung cancer (2). In China, the incidence of CRC ranks fourth among all malignancies and the mortality ranks fifth (3). Advances in treatment methods such as surgery, chemoradiotherapy, immunotherapy, and targeted therapy have improved the prognosis of CRC; however, many CRC patients experience distant metastasis or local recurrence after receiving anticancer therapy (4–7). The survival rate of CRC patients with recurrence and metastasis can be reduced to 5–10% (8, 9). Therefore, there is an urgent need to identify effective prognostic indicators to help clinicians design optimal treatment strategies.

Serum carcinoembryonic antigen (CEA) is a simple and common tumor marker for predicting the prognosis of CRC patients that is produced and released during tumorigenesis and development. CEA is an effective marker to assess the prognosis of CRC patients, and high levels of CEA may indicate more aggressive tumor characteristics and worse prognosis (10–12). Persistently elevated levels of CEA may suggest that patients are at high risk for recurrence and metastasis (13, 14). However, the specificity of serum CEA in CRC patients is not high, and only 40–50% of CRC patients have elevated serum CEA (15, 16). Therefore, it is necessary to integrate multiple indicators for the prognostic evaluation of CRC patients. The establishment of non-invasive prognostic predictors of cancer from anthropometric and serological markers has recently attracted widespread interest (17, 18). Increasing evidence suggests that the nutritional status of the host is closely related to disease progression (19–21). Perioperative malnutrition greatly increases the incidence of postoperative complications and is one of the main reasons for poor treatment outcomes. In 2005, Bouillanne et al. established a geriatric nutritional risk index (GNRI) based on common serological nutritional parameters and anthropometric parameters (22) that can be used as a nutritional prognostic assessment tool for hospitalized patients. GNRI is a simple, cost-effective, and accessible biomarker, and it has increasingly been used in the prognostic evaluation of various malignancies (23–26).

The predictive performance of a single prognostic indicator remains low, and the combination of multiple prognostic indicators may improve the predictive performance. The efficacy of the combination of GNRI and CEA for predicting the prognosis of CRC patients has not been investigated to date. In this study, we explore the value of the combination of GNRI and CEA for the prognosis assessment of CRC patients and provide references for the formulation of treatment strategies for CRC patients in clinical practice.

## Patients and Methods

### Population

This study retrospectively analyzed the medical records of CRC patients who underwent surgical treatment at the Colorectal and Anal Surgery Department of the First Affiliated Hospital of Guangxi Medical University between January 2012 and December 2015. The inclusion criteria were as follows: (1) age > 18 years and volunteered to participate in the study; (2) undergoing radical surgery for treatment purposes; (3) pathologically confirmed primary CRC; (4) complete preoperative clinicopathological characteristics. The exclusion criteria were as follows: (1) received neoadjuvant chemoradiotherapy before surgery; (2) received emergency surgery or received palliative resection; (3) multiple primary cancers. Patient information was collected from hospital medical record systems and was used only for research purposes. Patient information was anonymous during the analysis. The study was approved by the Institutional Ethics Review Board of our center, approval number: 2021 (KY-E-043).

### Demographics and Clinical Characteristics

The clinicopathological characteristics of the patients were systematically reviewed from the hospital’s medical records. Baseline data included sex, age, height, weight, and comorbidities (hypertension and diabetes). Preoperative serological data included white blood cell count, hemoglobin, neutrophil count, lymphocyte count, albumin, and serum CEA level. Fasting blood samples from CRC patients were collected 1 week before surgery and tested in the hospital laboratory. The pathological parameters included T stage, N stage, distant metastasis, tumor-node-metastasis (TNM) stage, perineural invasion, vascular invasion, macroscopic type, differentiation, tumor location, and tumor size. All pathological data were obtained from the evaluation of excised tissue samples by professional pathologists. The TNM stage was determined according to the eighth edition of the Union for International Cancer Control (UICC) pathology classification. Surgical information included surgical approach (laparoscopic or open). Body mass index (BMI) was defined as weight (kg)/square height (m^2^) (low: < 18.5, normal: 18.5–24, high: ≥ 24).

### Construction of the Geriatric Nutritional Risk Index-Carcinoembryonic Antigen Score

The GNRI formula was described previously (22, 24). GNRI was defined as follows: 1.487 × serum albumin concentration (g/L) + 41.7 × preoperative body weight (PBW)/ideal body weight (IBW). In this study, the IBW was defined according to the modified Broca index. When the PBW of patients exceeded the IBW, the PBW/IBW was set to 1. Patients were divided into three groups according to the GNRI as follows: normal (≥98), low risk (92–98), and high risk (<92). Serum CEA < 5.00 ng/ml was considered normal, and CEA ≥ 5.00 ng/ml was considered high. The GNRI-CEA score was assigned according to the following rules: for GNRI, a score of 1 was considered normal, 2 was considered low risk, and 3 was considered high risk; for CEA, a score of 1 was normal and 2 was high. The GNRI-CEA score was the sum of the two scores. Patients were divided into four groups according to the GNRI-CEA score: normal, mild, moderate, and severe (Supplementary Figure 1).

### Follow-Up and Outcomes

The survival status of patients was obtained from outpatient clinics or telephone calls. All patients were subjected to the same follow-up protocol, which included outpatient physical examinations every 3 months for 2 years and every 6 months after primary tumor resection. The physical examinations included serological testing, imaging examination, and colonoscopy. Postoperative follow-up was performed by professionally trained physicians. The last follow-up date was February 04, 2021. Survival outcomes included overall survival (OS) and progression-free survival (PFS). The start date for survival assessment was the date of surgery for the primary cancer. The date of recurrence was defined as the date on which local recurrence or distant metastasis was first confirmed by tissue biopsy, additional surgery, and/or radiographic imaging in CRC patients undergoing surgical treatment. PFS was defined as the time interval from tumor resection to first recurrence, death, or last follow-up. OS was defined as the time interval between tumor resection and death or last follow-up.

### Statistical Analysis

The Pearson Chi-square test or Fisher’s exact test was used for comparison of categorical variables, and Student’s *t*-test was used for continuous variables. Restricted cubic spline was used to assess the dose-response relationship between factors (GNRI and CEA) and survival in CRC patients. Survival analysis was performed by the Kaplan-Meier curve and log-rank test. Cox proportional hazards regression analysis was used to assess risk factors associated with PFS and OS. The discriminant indices, including Harrell’s concordance index (C-index), continuous net reclassification improvement (cNRI), and integrated discrimination improvement (IDI) were calculated to assess and compare the discrimination capacity of the predictors to predict mortality. The R package “survival” was used to construct prognostic nomograms to predict 1–5 year PFS and OS in CRC patients. The C-index and calibration curve were used to evaluate the prognostic accuracy of the nomograms. Receiver operating characteristic (ROC) curve analysis was performed to compare the prognostic predictive ability of the nomograms with pathological stages. The patient population was randomly divided into two internal validation datasets at a ratio of 7:3 to evaluate the generalizability of the nomograms. Two-sided *p* < 0.05 was considered statistically significant. All parameters were analyzed by SPSS 24.0 (IBMSPSS, IBM CorPoration, Armonk, NY) and R software (4.0.2; R Foundation for Statistical Computing, Vienna, Austria).

## Results

### Patient Characteristics

A total of 1,014 CRC patients who underwent radical resection were included in the study. There were 639 (63.0%) men and 375 (37.0%) women. The mean age was **57.33** ± 13.34 years. There were 184 (18.1%) patients in stage I, 328 (32.3%) patients in stage II, 397 (39.2%) patients in stage III, and 105 (10.4%) patients in stage IV. There were 586 CRC patients with normal CEA and 428 CRC with high CEA. CRC patients with normal, low, and high risk GNRI were 243, 303, and 468, respectively (Supplementary Table 1). Normal, mild, moderate, and severe GNRI-CEA scores were 287, 355, 254, and 118, respectively. There was no significant correlation between serum CEA and GNRI (*p* = 0.801; Spearman’s correlation coefficient, *r*s = 0.063) (Supplementary Figure 2). Comparison of the clinicopathological characteristics among the GNRI-CEA score groups showed that the GNRI-CEA score was closely associated with male gender, advanced age, low BMI, advanced T stage, metastasis, advanced pathological stage, colon cancer, large tumor size, low hemoglobin, high neutrophil count, low lymphocyte count, and low albumin. In addition, CRC patients with severe GNRI-CEA score had a longer hospital stay by approximately 3 days, a higher risk of recurrence and metastasis, and a higher risk of death. The details of the association between clinicopathological characteristics and GNRI-CEA score are shown in Table 1.

**TABLE 1:** The relationships between the GNRI-CEA score and clinicopathological factors of CRC patients.

Characteristic	GNRI-CEA score				*p*
	Normal, *n* = 287	Mild, *n* = 355	Moderate, *n* = 254	Severe, *n* = 118	
Gender, male, n (%)	160 (55.7)	230 (64.8)	168 (66.1)	81 (68.6)	0.022
Age, years, mean (SD)	53.48 (12.50)	57.64 (12.60)	59.65 (13.88)	60.81 (14.19)	<0.001
BMI, kg/m^2^, mean (SD)	23.21 (2.93)	22.78 (3.02)	20.80 (3.36)	20.31 (3.51)	<0.001
Hypertension, yes, n (%)	37 (12.9)	50 (14.1)	41 (16.1)	25 (21.2)	0.171
Diabetes, yes, n (%)	13 (4.5)	25 (7.0)	16 (6.3)	11 (9.3)	0.308
T stage, T3–4, n (%)	191 (66.6)	280 (78.9)	189 (74.4)	105 (89.0)	<0.001
N stage, n (%)					0.995
N0	159 (55.4)	191 (53.8)	136 (53.5)	63 (53.4)	
N1	81 (28.2)	106 (29.9)	75 (29.5)	33 (28.0)	
N2	47 (16.4)	58 (16.3)	43 (16.9)	22 (18.6)	
Metastasis, yes, n (%)	10 (3.5)	34 (9.6)	29 (11.4)	32 (27.1)	<0.001
TNM stage, n (%)					<0.001
Stage I	68 (23.7)	54 (15.2)	53 (20.9)	9 (7.6)	
Stage II	87 (30.3)	127 (35.8)	76 (29.9)	38 (32.2)	
Stage III	122 (42.5)	140 (39.4)	96 (37.8)	39 (33.1)	
Stage IV	10 (3.5)	34 (9.6)	29 (11.4)	32 (27.1)	
Tumor location, rectal, n (%)	168 (58.5)	183 (51.5)	107 (42.1)	46 (39.0)	<0.001
Tumor size [median (IQR)]	4.00 (2.00)	4.50 (2.10)	5.00 (2.50)	6.00 (2.50)	<0.001
Perineural invasion, positive, n (%)	24 (8.4)	32 (9.0)	22 (8.7)	12 (10.2)	0.948
Vascular invasion, positive, n (%)	41 (14.3)	56 (15.8)	37 (14.6)	17 (14.4)	0.951
Macroscopic type, n (%)					0.870
Protrude type	69 (24.0)	85 (23.9)	64 (25.2)	32 (27.1)	
Infiltrating type	22 (7.7)	36 (10.1)	27 (10.6)	10 (8.5)	
Ulcerative type	196 (68.3)	234 (65.9)	163 (64.2)	76 (64.4)	
Differentiation, poor, n (%)	38 (13.2)	38 (10.7)	32 (12.6)	15 (12.7)	0.777
White blood cell [median (IQR)]	6.44 (2.55)	6.70 (2.23)	6.82 (2.87)	6.96 (3.21)	0.153
Hemoglobin [median (IQR)]	126.00 (21.60)	120.10 (26.5)	112.45 (33.72)	104.25 (32.88)	<0.001
Neutrophil [median (IQR)]	3.72 (1.60)	3.81 (1.72)	4.13 (2.29)	4.46 (2.97)	<0.001
Lymphocyte [median (IQR)]	1.94 (0.82)	1.81 (0.77)	1.60 (0.71)	1.48 (0.72)	<0.001
Albumin [median (IQR)]	40.80 (3.25)	38.70 (4.20)	35.55 (3.70)	32.75 (3.87)	<0.001
Length of stay [median (IQR)]	18.00 (6.50)	18.00 (7.00)	19.00 (7.00)	21.00 (7.00)	<0.001
Recurrence and metastasis, yes, n (%)	58 (20.2)	96 (27.0)	92 (36.2)	51 (43.2)	<0.001
Death, yes, n (%)	83 (28.9)	141 (39.7)	141 (55.5)	79 (66.9)	<0.001

### The Association of Geriatric Nutritional Risk Index, Carcinoembryonic Antigen, and Geriatric Nutritional Risk Index-Carcinoembryonic Antigen Score and Survival

**FIGURE 1:**
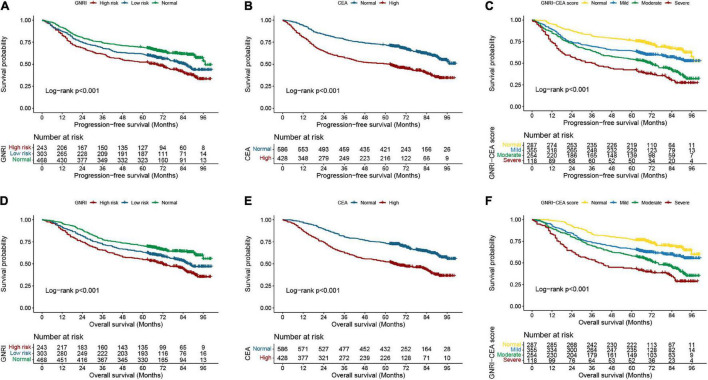
Kaplan-Meier curve of GNRI, CEA, and GNRI-CEA score in CRC patients. **(A)** PFS of GNRI; **(B)** PFS of CEA; **(C)** PFS of GNRI-CEA score; **(D)** OS of GNRI; **(E)** OS of CEA; OS of GNRI-CEA score; **(F)** OS of GNRI-CEA score.

A total of 297 (29.29%) CRC patients developed recurrence and metastasis. PFS was lower in patients with high risk GNRI than in those with low-risk/normal GNRI (39.92% vs. 51.16% vs. 62.61%, *p* < 0.001) (Figure 1A). Patients with high CEA had significantly worse PFS than those with normal CEA (41.36% vs. 62.80%, *p* < 0.001) (Figure 1B). The GNRI-CEA score was used to further stratify the prognosis of CRC patients; the PFS of patients showed a step-like decrease from the normal group to the severe group (68.99% vs. 57.75% vs. 41.34% vs. 31.36%, *p* < 0.001) (Figure 1C). During the follow-up period, 444 (43.79%) patients died. Patients with high risk GNRI had worse OS than those with low-risk/normal GNRI (42.39% vs. 53.80% vs. 64.96%, *p* < 0.001) (Figure 1D). The OS was lower in the high CEA group than in the normal CEA group (43.93% vs. 65.19%, *p* < 0.001) (Figure 1E). Compared with patients with a normal GNRI-CEA score, patients with mild/moderate/severe GNRI-CEA scores had a significantly lower OS (68.99% vs. 57.75% vs. 41.34% vs. 31.36%, *p* < 0.001) (Figure 1F).

**FIGURE 2:**
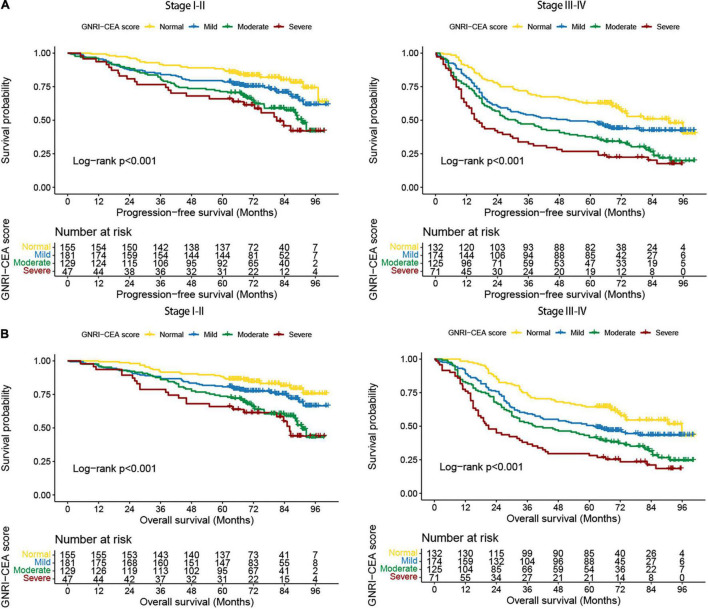
Stratified survival analysis of GNRI-CEA score based on TNM stage. **(A)** PFS; **(B)** OS.

We performed a stratified survival analysis for PFS and OS based on TNM stage. Regardless of early stage or advanced stage, the PFS and OS were significantly lower in patients with high-risk GNRI than in those with normal GNRI (Supplementary Figures 3A,B), and the PFS and OS were also significantly lower in patients with high CEA than in those with normal CEA (Supplementary Figures 4A,B). The GNRI-CEA score was still able to effectively stratify the prognosis of CRC patients at different pathological stages. The PFS and OS of the severe group were significantly poorer than those of the mild/moderate/severe groups (Figures 2A,B).

### Prognostic Factors in Colorectal Cancer Patients

**FIGURE 3:**
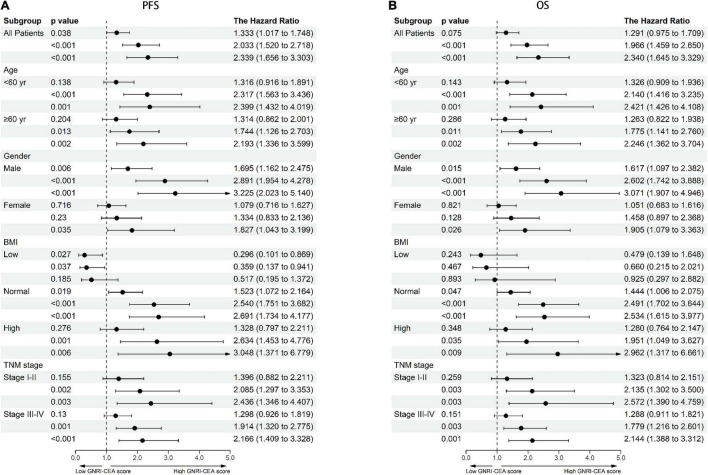
Dose-response effects of GNRI-CEA score based on subgroup **(A)** PFS, **(B)** OS. Adjusted for gender, age, BMI, hypertension, diabetes, T stage, N stage, metastasis, tumor location, tumor size, perineural invasion, vascular invasion, macroscopic type, differentiation.

There was a clear dose-response relationship between GNRI/CEA and survival in CRC patients under different adjustment models. GNRI was positively correlated with survival (Supplementary Figures 5A,B), and CEA was negatively correlated with survival (Supplementary Figures 5C,D). After adjusting for confounders, low GNRI and high CEA were risk factors affecting most subgroups of CRC patients (Supplementary Figures 6, 7). An increase in the GNRI-CEA score was associated with a progressively poorer prognosis of CRC patients. Compared with the normal group, the severe group had a more than twofold higher risk of adverse outcomes regarding both PFS [hazard ratio (HR), 2.339; 95% confidence interval (CI), 1.656–3.303; *p* < 0.001) and OS (HR, 2.340; 95% CI, 1.645–3.329; *p* < 0.001) (Tables 2, 3)]. In subgroup analysis, we found that the GNRI-CEA score showed a significant prognostic dose-response in PFS (Figure 3A) and OS (Figure 3B). We further compared the discriminative ability of GNRI, CEA, and GNRI-CEA score for evaluating the prognosis of CRC patients. Compared with other indices, the GNRI-CEA score had good prognostic prediction ability for both PFS (Supplementary Table 2) and OS (Supplementary Table 3).

**TABLE 2:** Trend test of the relationship between GNRI/CEA and progression-free survival.

	Model a	*p*-value	Model b	*p*-value	Model c	*p*-value
**GNRI**						
Continuous (per SD)	0.814 (0.75, 0.883)	<0.001	0.849 (0.773, 0.934)	0.001	0.88 (0.798, 0.97)	0.010
Cutoff value		<0.001		<0.001		<0.001
High risk	ref		ref		ref	
Low risk	0.767 (0.61, 0.964)	0.023	0.816 (0.64, 1.04)	0.1	0.829 (0.645, 1.064)	0.141
Normal	0.563 (0.452, 0.702)	<0.001	0.625 (0.486, 0.803)	<0.001	0.588 (0.451, 0.768)	<0.001
*Quartiles*						
Q1	ref		ref		ref	
Q2	0.815 (0.644, 1.031)	0.088	0.864 (0.674, 1.108)	0.25	0.894 (0.693, 1.154)	0.391
Q3	0.599 (0.467, 0.77)	<0.001	0.655 (0.497, 0.864)	0.003	0.61 (0.457, 0.813)	0.001
Q4	0.529 (0.405, 0.69)	<0.001	0.588 (0.438, 0.79)	<0.001	0.555 (0.406, 0.758)	<0.001
p for trend		<0.001		<0.001		<0.001
**CEA**						
Continuous (per SD)	1.276 (1.147, 1.419)	<0.001	1.28 (1.149, 1.427)	<0.001	1.176 (1.054, 1.313)	0.004
Cutoff value						
Normal	ref		ref		ref	
High	2.017 (1.682, 2.42)	<0.001	1.997 (1.663, 2.399)	<0.001	1.499 (1.233, 1.822)	<0.001
p for trend		<0.001		<0.001		<0.001
*Quartiles*						
Q1	ref		ref		ref	
Q2	1.553 (1.154, 2.09)	0.004	1.56 (1.158, 2.101)	0.003	1.507 (1.114, 2.039)	0.008
Q3	2.102 (1.578, 2.8)	<0.001	2.084 (1.558, 2.788)	<0.001	1.713 (1.271, 2.309)	<0.001
Q4	3.026 (2.292, 3.996)	<0.001	3.039 (2.298, 4.018)	<0.001	1.984 (1.477, 2.664)	<0.001
p for trend		<0.001		0.001		<0.001
**GNRI-CEA score**						
Normal	ref		ref		ref	
Mild	1.529 (1.176, 1.988)	0.002	1.522 (1.169, 1.981)	0.002	1.333 (1.017, 1.748)	0.038
Moderate	2.207 (1.697, 2.87)	<0.001	2.145 (1.626, 2.831)	<0.001	2.033 (1.52, 2.718)	<0.001
Severe	3.093 (2.288, 4.181)	<0.001	2.986 (2.165, 4.118)	<0.001	2.339 (1.656, 3.303)	<0.001
p for trend		<0.001		<0.001		<0.001

*Model a: No adjusted.*

*Model b: Adjusted for gender, age, and BMI.*

*Model c: Adjusted for gender, age, BMI, hypertension, diabetes, T stage, N stage, metastasis, tumor location, tumor size, perineural invasion, vascular invasion, macroscopic type, differentiation.*

**TABLE 3:** Trend test of the relationship between GNRI/CEA and overall survival.

	Model a	*p*-value	Model b	*p*-value	Model c	*p*-value
**GNRI**						
Continuous (per SD)	0.808 (0.743, 0.878)	<0.001	0.844 (0.767, 0.929)	0.001	0.881 (0.798, 0.973)	0.013
Cutoff value		<0.001		<0.001		<0.001
High risk	ref		ref		ref	
Low risk	0.758 (0.6, 0.958)	0.021	0.803 (0.627, 1.029)	0.082	0.829 (0.642, 1.07)	0.15
Normal	0.549 (0.438, 0.688)	<0.001	0.61 (0.472, 0.789)	<0.001	0.58 (0.442, 0.762)	<0.001
*Quartiles*						
Q1	ref		ref		ref	
Q2	0.812 (0.638, 1.032)	0.089	0.859 (0.667, 1.106)	0.238	0.906 (0.699, 1.175)	0.459
Q3	0.565 (0.436, 0.733)	<0.001	0.618 (0.465, 0.821)	0.001	0.572 (0.425, 0.769)	<0.001
Q4	0.525 (0.4, 0.689)	<0.001	0.584 (0.432, 0.79)	<0.001	0.563 (0.409, 0.774)	<0.001
p for trend		<0.001		<0.001		<0.001
**CEA**						
Continuous (per SD)	1.269 (1.138, 1.415)	<0.001	1.272 (1.138, 1.421)	<0.001	1.179 (1.053, 1.319)	0.004
*Cutoff value*						
Normal	ref		ref		ref	
High	2.034 (1.687, 2.453)	<0.001	2.003 (1.659, 2.417)	<0.001	1.475 (1.207, 1.803)	<0.001
p for trend		<0.001		<0.001		<0.001
*Quartiles*						
Q1	ref		ref		ref	
Q2	1.446 (1.064, 1.965)	0.019	1.439 (1.058, 1.957)	0.02	1.392 (1.018, 1.902)	0.038
Q3	2.028 (1.512, 2.72)	<0.001	1.983 (1.473, 2.669)	<0.001	1.598 (1.178, 2.169)	0.003
Q4	3.004 (2.263, 3.988)	<0.001	2.991 (2.251, 3.976)	<0.001	1.92 (1.422, 2.592)	<0.001
p for trend		<0.001		0.001		<0.001
**GNRI-CEA score**						
*Normal*						
Mild	1.531 (1.167, 2.007)	0.002	1.512 (1.151, 1.986)	0.003	1.291 (0.975, 1.709)	0.075
Moderate	2.2 (1.678, 2.886)	<0.001	2.123 (1.595, 2.825)	<0.001	1.966 (1.459, 2.65)	<0.001
Severe	3.258 (2.394, 4.436)	<0.001	3.111 (2.24, 4.321)	<0.001	2.340 (1.645, 3.329)	<0.001
p for trend		<0.001		<0.001		<0.001

*Model a: No adjusted.*

*Model b: Adjusted for gender, age, and BMI.*

*Model c: Adjusted for gender, age, BMI, hypertension, diabetes, T stage, N stage, metastasis, tumor location, tumor size, perineural invasion, vascular invasion, macroscopic type, differentiation.*

### Construction of Prognostic Prediction Nomograms Based on Geriatric Nutritional Risk Index-Carcinoembryonic Antigen Score

**FIGURE 4:**
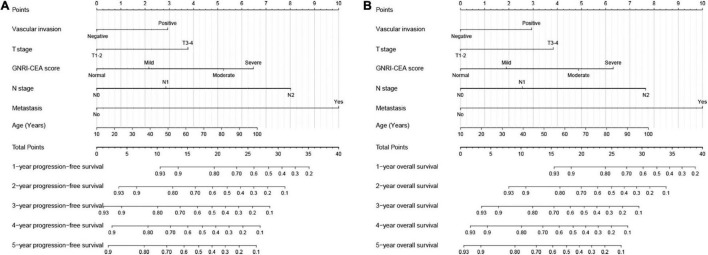
Construction the novel prognostic nomograms in CRC patients. **(A)** The PFS nomogram; **(B)** the OS nomogram.

Univariate COX regression survival analysis showed that the following clinicopathological features were significantly associated with prognosis: age, BMI, T stage, pathological N stage, distant metastasis, tumor size, perineural invasion, vascular invasion, differentiation, surgical approach, and GNRI-CEA score. However, in the multivariate analysis, only age (HR, 1.010; 95% CI, 1.002–1.017; *p* = 0.008), pathological T stage (T3–4) (HR, 1.590; 95% CI, 1.208–2.093; *p* = 0.001), high N stage (*p* < 0.001), distant metastasis (HR, 3.361; 95% CI, 2.615–4.319; *p* < 0.001), vascular invasion (HR, 1.419; 95% CI, 1.106–1.821; *p* = 0.006) and high GNRI-CEA score (*p* < 0.001) were independent risk factors for PFS in CRC patients (Supplementary Table 4). Similarly, only age (HR, 1.012; 95% CI, 1.005–1.019; *p* = 0.001), pathological T stage (T3–4) (HR, 1.626; 95% CI, 1.221–2.165; *p* = 0.001), high N stage (*p* < 0.001), distant metastasis (HR, 3.579; 95% CI, 2.779–4.608; *p* < 0.001), vascular invasion (HR, 1.468; 95% CI, 1.14–1.891; *p* = 0.003) and high GNRI-CEA score (*p* < 0.001) were independent risk factors for OS in CRC patients (Supplementary Table 5). Based on the prognostic variables identified in the multivariate analysis, we developed two survival nomograms to predict 1–5-year PFS (Figure 4A) and 1–5-year OS (Figure 4B) in CRC patients.

### Utility Evaluation of the Nomograms

The C-indexes of the PFS and OS nomograms were 0.733 (95% CI, 0.709–0.757) and 0.726 (95% CI, 0.703–0.749), respectively. The 3- and 5-year calibration curves demonstrated the best agreement between predicted survival probabilities and actual observed probabilities (Supplementary Figures 8A,B). Compared with traditional TNM staging, the present nomogram had better resolution and accuracy for predicting 3- and 5-year PFS (Supplementary Figure 9A) and OS (Supplementary Figure 9B). The results of time-dependent ROC curves demonstrated that the developed nomograms predicted the prognosis of CRC patients with high accuracy (Supplementary Figure 10A). Next, CRC patients were divided into low-risk and high-risk groups according to the median nomogram score, and the results showed that CRC patients in the high-risk group had significantly poorer PFS/OS than those in the low-risk group (Supplementary Figure 10B).

### Randomized Internal Validation of the Nomograms

The study population was then divided into validation a (712) and validation b (302) groups in a ratio of 7:3 for randomized internal validation. In validation a, the PFS and OS C-index values were 0.732 (0.705–0.759) and 0.734 (0.706–0.762), respectively, whereas those in validation b were 0.723 (0.683–0.763) and 0.740 (0.697–0.783), respectively. Calibration curves demonstrated the best agreement between predicted survival probabilities and actual observations in both validation a (Supplementary Figure 11A) and validation b (Supplementary Figure 11B) groups. In the validation cohorts, the developed nomograms were superior to TNM staging in predicting the prognosis of CRC patients (Supplementary Figures 11C,D). The present nomogram also achieved good predictive accuracy (Supplementary Figure 12A) and prognostic discrimination (Supplementary Figure 12B) in the internal validation cohorts.

## Discussion

In this study, we showed that GNRI is an effective factor for predicting the prognosis of CRC patients. High-risk GNRI patients had an approximately 1.7-fold higher risk of poor prognosis than normal GNRI patients. GNRI combines body weight changes and serological nutritional markers and is a promising nutritional index for predicting the prognosis of cancer. Malnutrition is an important factor affecting the prognosis of cancer patients, especially those with gastrointestinal cancer. Malnutrition is a common occurrence in cancer and is present in 34–71% of cancer patients due to the tumor itself or various anticancer treatments (27, 28). Malnutrition has a huge negative impact on the clinical outcomes of cancer patients. Malnutrition is associated with a higher incidence of postoperative infections and complications and may reduce quality of life, increase chemotherapy toxicity, or delay treatment, resulting in shorter survival, longer hospital stays, and increased healthcare costs (29–33).

Doi et al. (34) found that a low GNRI score is associated with a higher risk of non-cancer mortality. However, the study was limited by a small number of samples, especially the small number of patients per tumor stage. In the present study, we analyzed a relatively large cohort and confirmed that GNRI is an independent factor affecting OS and PFS in CRC patients. Kato et al. (35) reported that low GNRI is independently associated with decreased survival in patients with early stage CRC aged ≥ 75 years. Ide et al. (36) showed that GNRI is an effective tool to identify locally advanced rectal cancer patients with a high risk of recurrence and improve the survival rate. In this study, we showed that GNRI can effectively differentiate patients at different pathological stages, whether early or advanced.

However, assessing only the host nutritional status to predict the prognosis of CRC patients has limitations because of the lack of tumor specificity. CEA is an effective marker for predicting the prognosis of CRC patients and is a commonly used monitoring index in the postoperative follow-up of CRC patients. In this study, we found that CEA is an independent prognostic factor affecting CRC patients, and the risk of poor prognosis was approximately 1.4-fold higher in patients with high CEA than in those with normal CEA. We combined GNRI and CEA to assess the prognosis of CRC patients and found that patients with low GNRI and high CEA had a more than twofold higher risk of death than those with both normal GNRI and CEA. The GNRI-CEA score can effectively stratify the prognosis of CRC patients and it is thus an effective supplement to assess the prognosis of patients with the same pathological stage. CEA indicates tumor burden, whereas GNRI indicates nutritional status. The GNRI-CEA score combines the advantages of the two and can accurately reflect the disease state of cancer patients, thereby effectively predicting prognosis. The present results suggest that a comprehensive analysis combining factors such as nutrition, tumor markers, and physical status could be effective in CRC prognostic studies. Although patients with a normal or high BMI appear healthy on preoperative examinations and are thus considered eligible for surgery, true malnutrition states and intolerable organ function are often overlooked. We found that the GNRI-CEA score could further stratify patients with normal or high BMI, suggesting that preoperative determination of the GNRI-CEA score in CRC patients can help identify truly malnourished patients.

The nomogram is a simple and effective tool for providing individualized risk predictions for patients. Here, we incorporated independent prognostic factors identified in multivariate analysis and developed nomograms to predict the clinical outcomes of CRC patients. These nomograms integrate individual profiles, tumor characteristics, serum tumor markers, and nutritional status, resulting in good prognostic predictive efficacy. The developed nomograms also achieved good predictive accuracy in the randomized internal validation cohorts. The results indicate that the developed nomograms have good reliability and accuracy, and can provide a personalized reference for prognosis assessment and clinical decision-making in CRC patients.

To the best of our knowledge, this study was the first to report the prognostic value of a combination of GNRI and CEA in CRC patients. In addition, we constructed novel prognostic nomograms to individually predict the survival of CRC patients, which have promising application potential in clinical practice. The present study had some limitations. Because of its retrospective nature, the potential for selection bias needs to be considered. Although this study performed randomized internal validation and confirmed that the novel nomograms have good predictive ability, the results need to be validated with external populations in the future.

## Conclusion

This study demonstrated that the combination of GNRI and CEA can effectively stratify the prognosis of CRC patients. The nomogram established based on the two indices can provide a personalized reference for prognosis assessment and clinical decision-making in CRC patients.

## Data Availability Statement

The datasets used and analyzed during the current study are available from the corresponding author on reasonable request.

## Ethics Statement

The studies involving human participants were reviewed and approved by the Ethics Committee of the First Affiliated Hospital of Guangxi Medical University: 2021 (KY-E-043). The patients/participants provided their written informed consent to participate in this study.

## Author Contributions

JG: conception and design. JG and ST: management support. HX, GY, and ML: data collection. YL, SG, QW, and XL: follow-up. HX: data analysis and professional drafting. HX and LW: manuscript writing. All authors agreed to publish and contributed to the article and approved the submitted version.

## Conflict of Interest

The authors declare that the research was conducted in the absence of any commercial or financial relationships that could be construed as a potential conflict of interest.

## Publisher’s Note

All claims expressed in this article are solely those of the authors and do not necessarily represent those of their affiliated organizations, or those of the publisher, the editors and the reviewers. Any product that may be evaluated in this article, or claim that may be made by its manufacturer, is not guaranteed or endorsed by the publisher.
