# Supplementary material for: Combination of Geriatric Nutritional Risk Index and Carcinoembryonic Antigen to Predict the Survival of Patients With Colorectal Cancer
Source: Front Nutr. 2022 Jun 30;9:902080. doi: 10.3389/fnut.2022.902080 (PMC9280638; doi:10.3389/fnut.2022.902080)
Supplement: Supplementary file 1 [file Data_Sheet_1.docx]

**Supplemental material**

**Figure S1.** Construction of GNRI-CEA score.

**
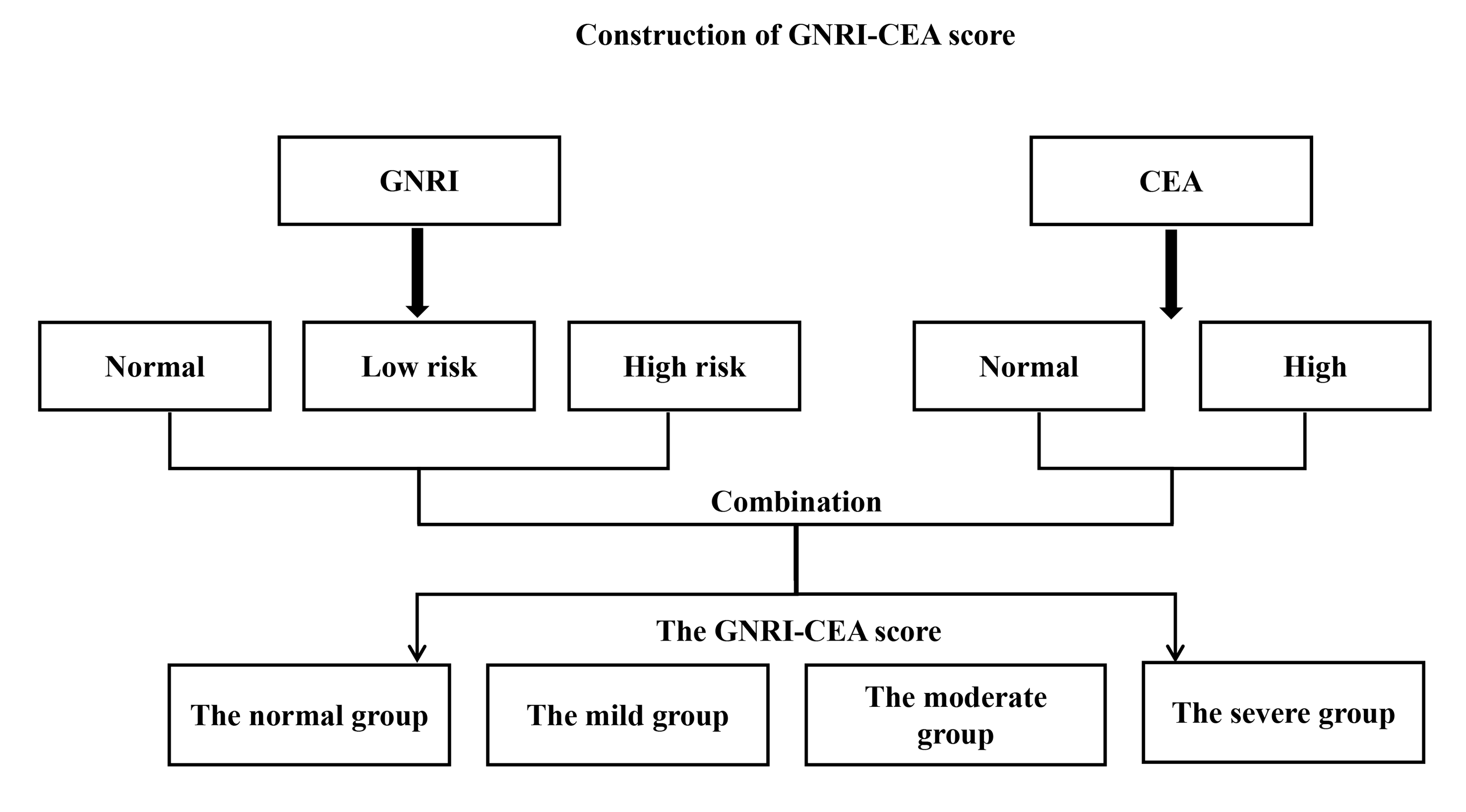
**

**Figure S2.** Correlations between CEA and GNRI.

**
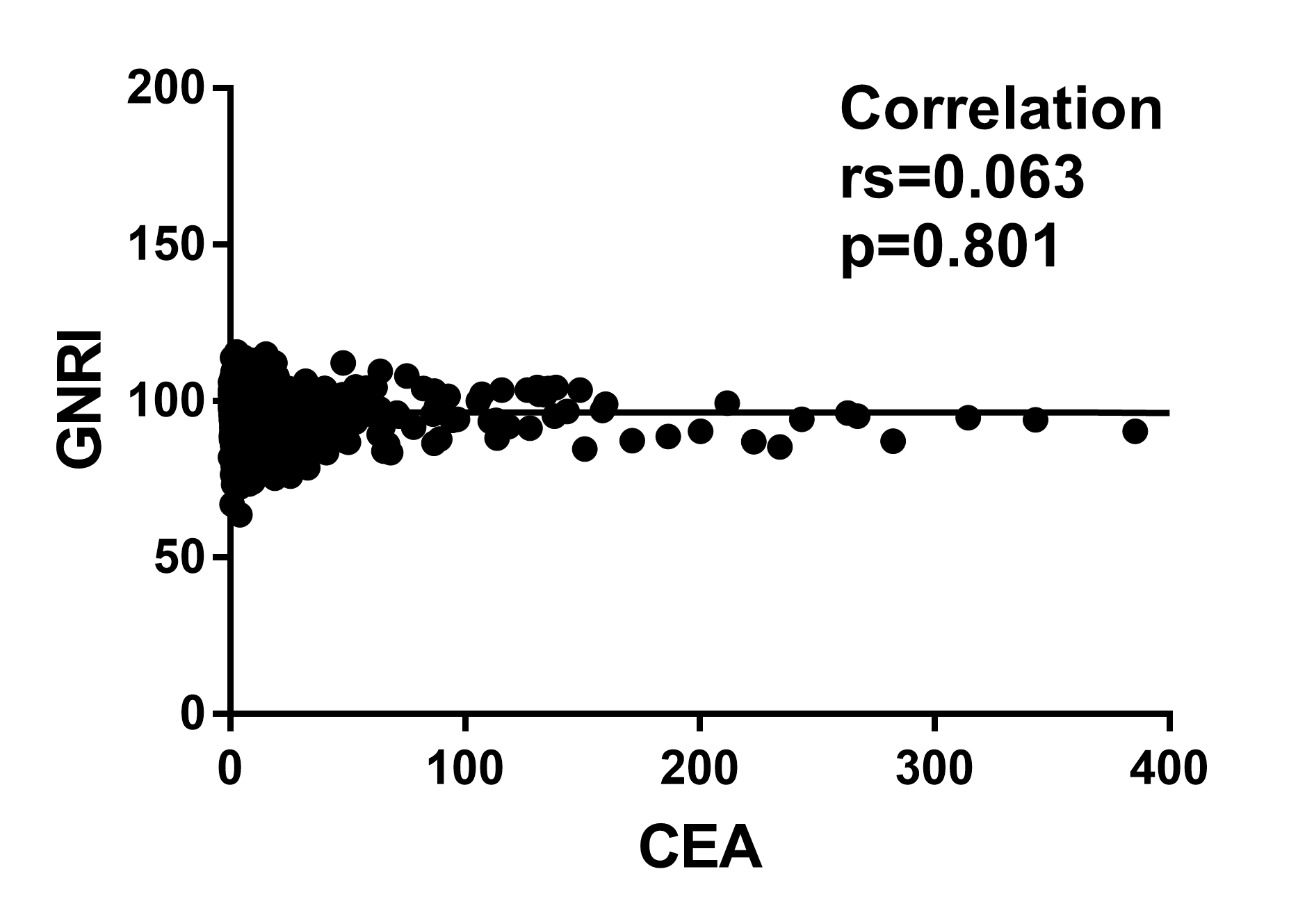
**

**Figure S3.** Stratified survival analysis of GNRI based on TNM stage.


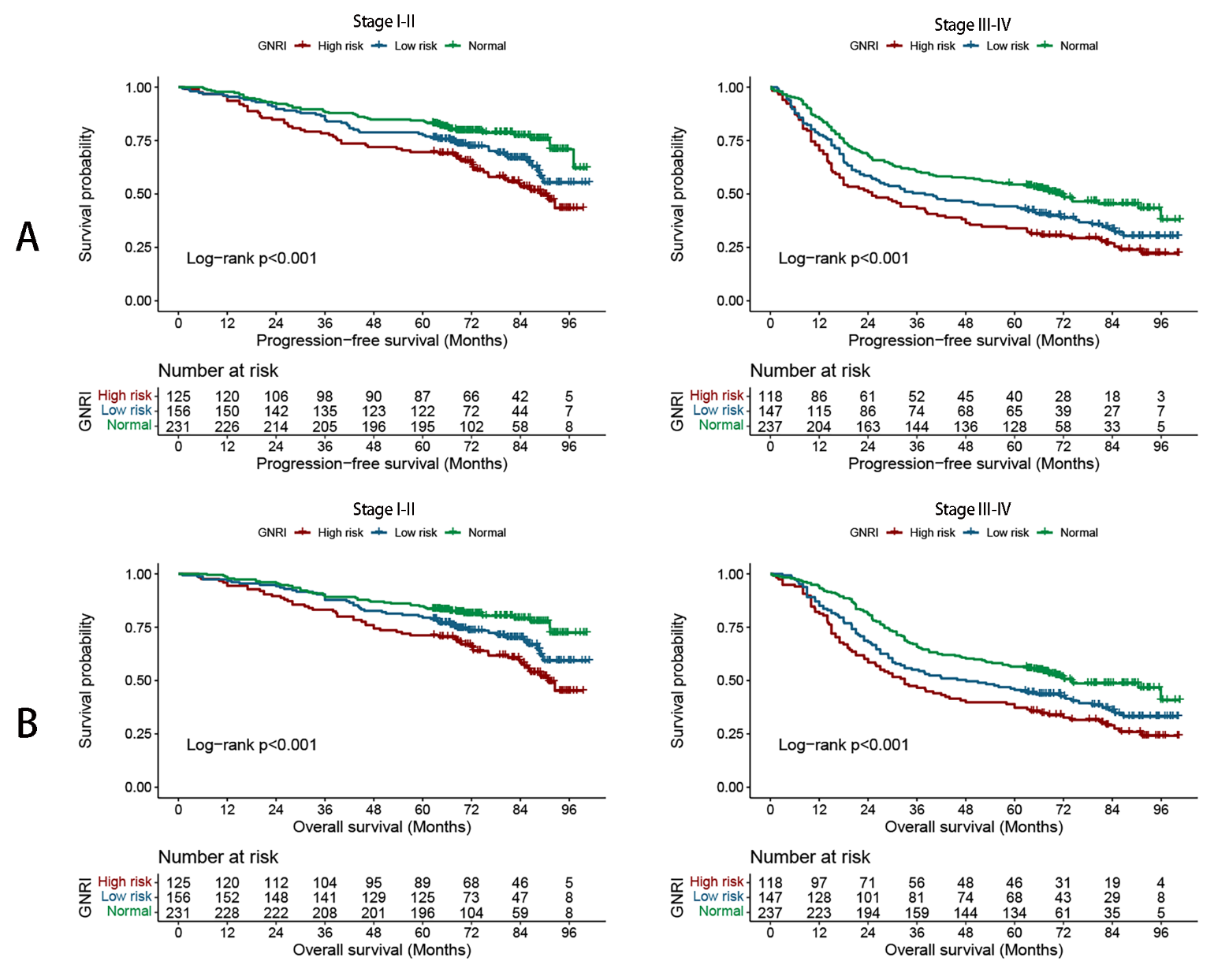


**Notes:** A, PFS; B, OS.

**Figure S4.** Stratified survival analysis of CEA based on TNM stage.


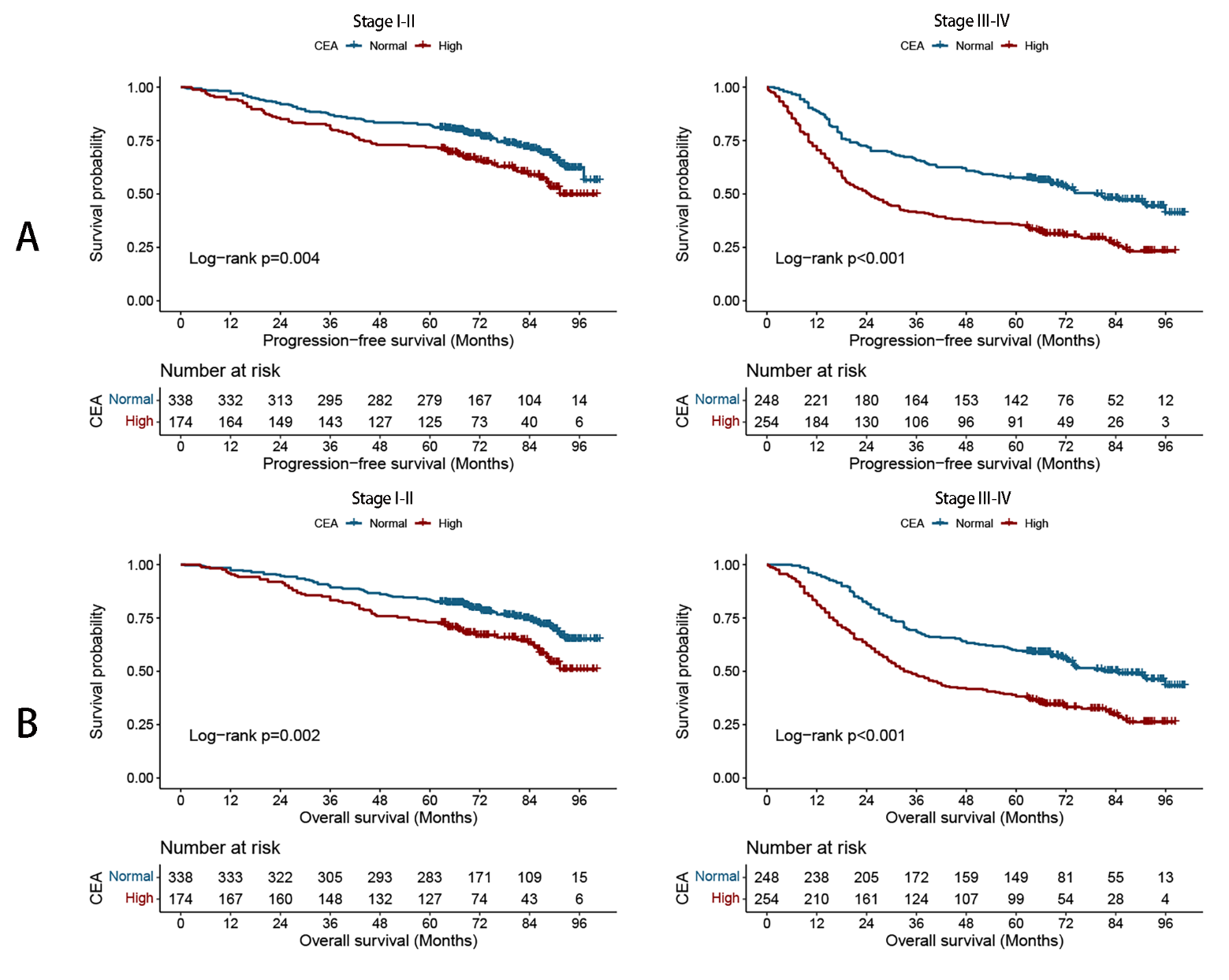


**Notes:** A, PFS; B, OS.

**Figure S5.** The association between GNRI/CEA and survival in CRC patients.
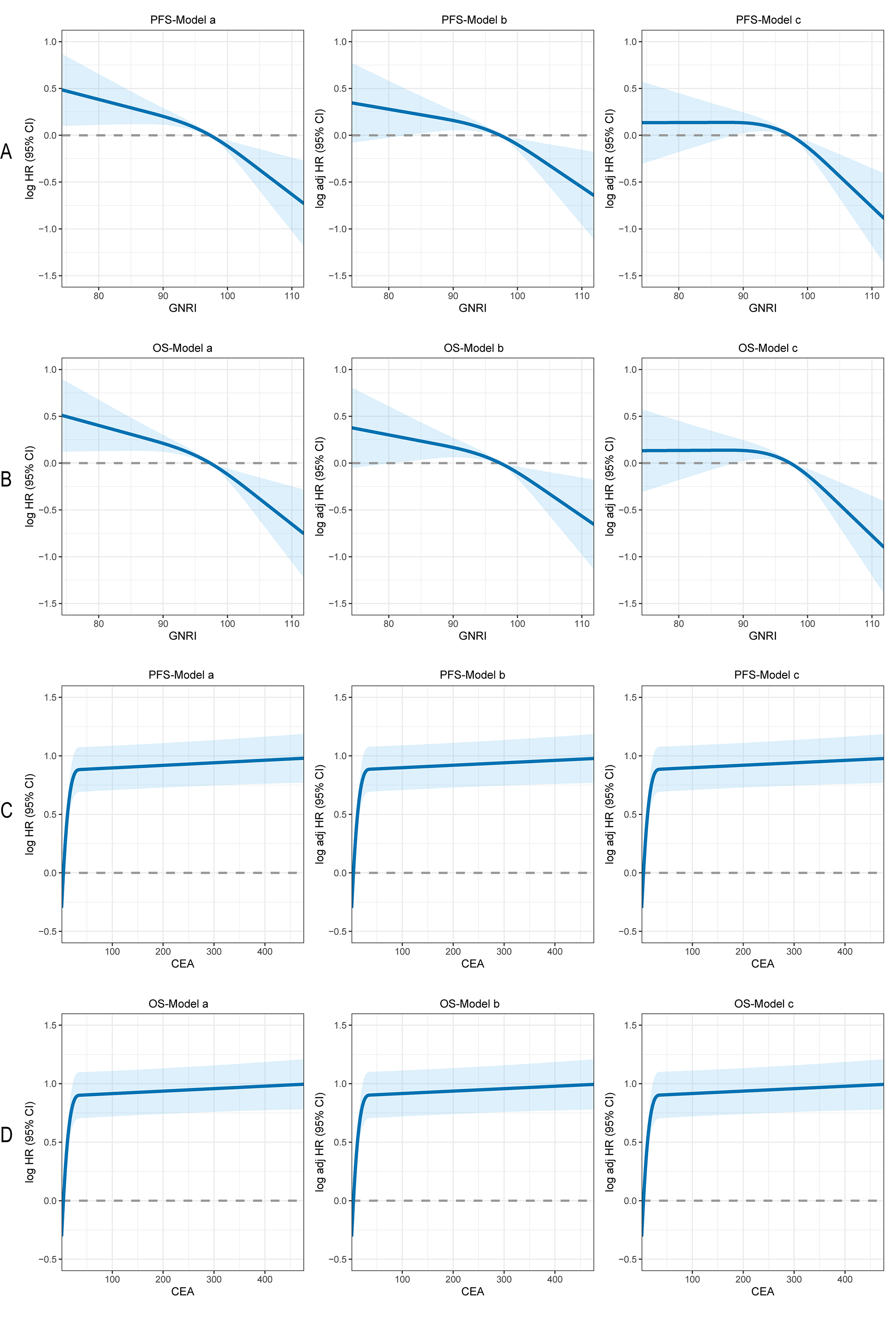


**Notes:** A, PFS of GNRI; B, OS of GNRI; C, PFS of CEA; D, OS of CEA;

Model a: No adjusted.

Model b: Adjusted for gender, age, and BMI.

Model c: Adjusted for gender, age, BMI, hypertension, diabetes, T stage, N stage, metastasis, tumor location, tumor size, perineural invasion, vascular invasion, macroscopic type, differentiation.

**Figure S6.** The association between GNRI and hazard risk of OS and PFS in various subgroups. (A,PFS; B, OS)


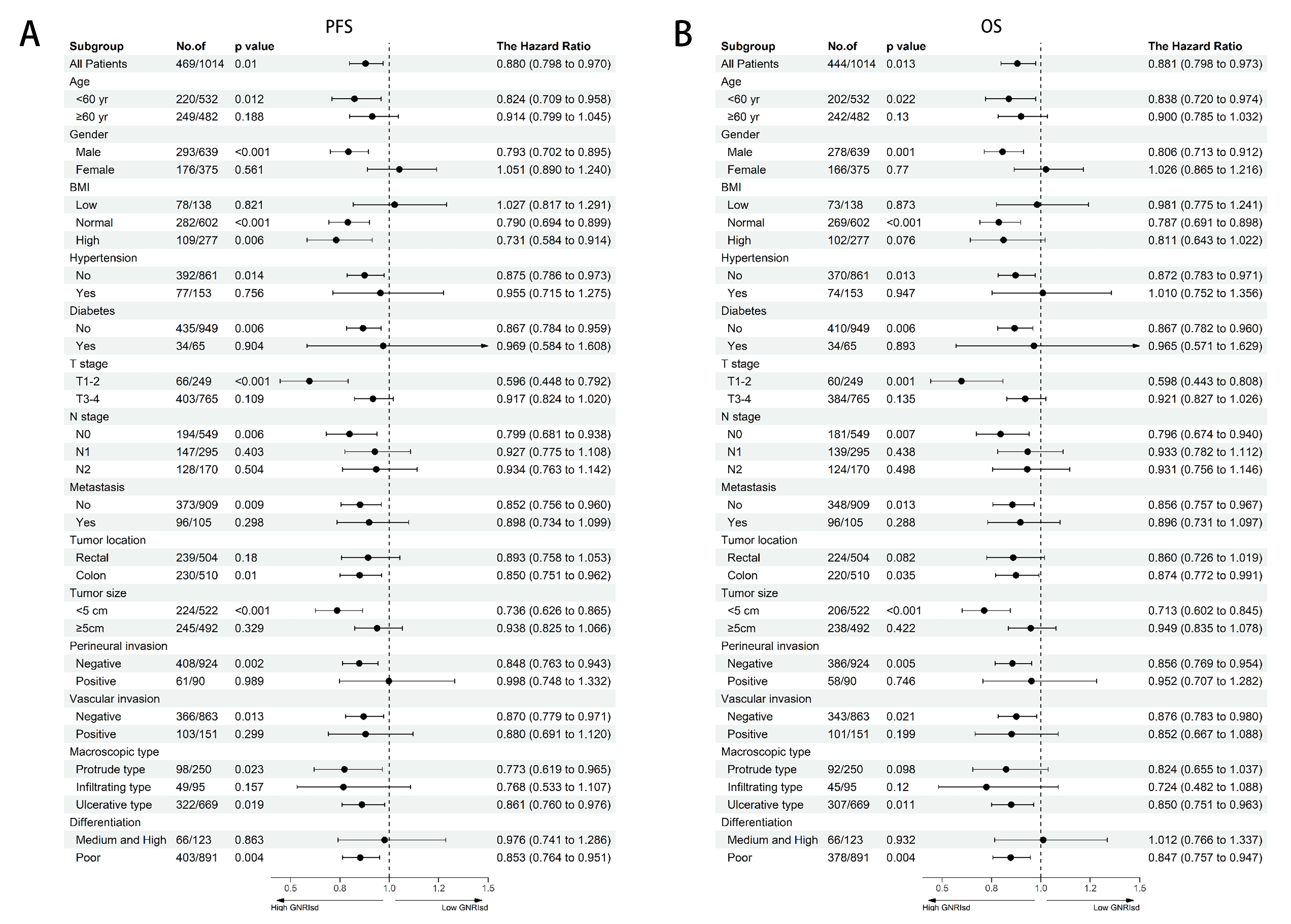


**Figure S7.** The association between CEA and hazard risk of OS and PFS in various subgroups. (A,PFS; B, OS)


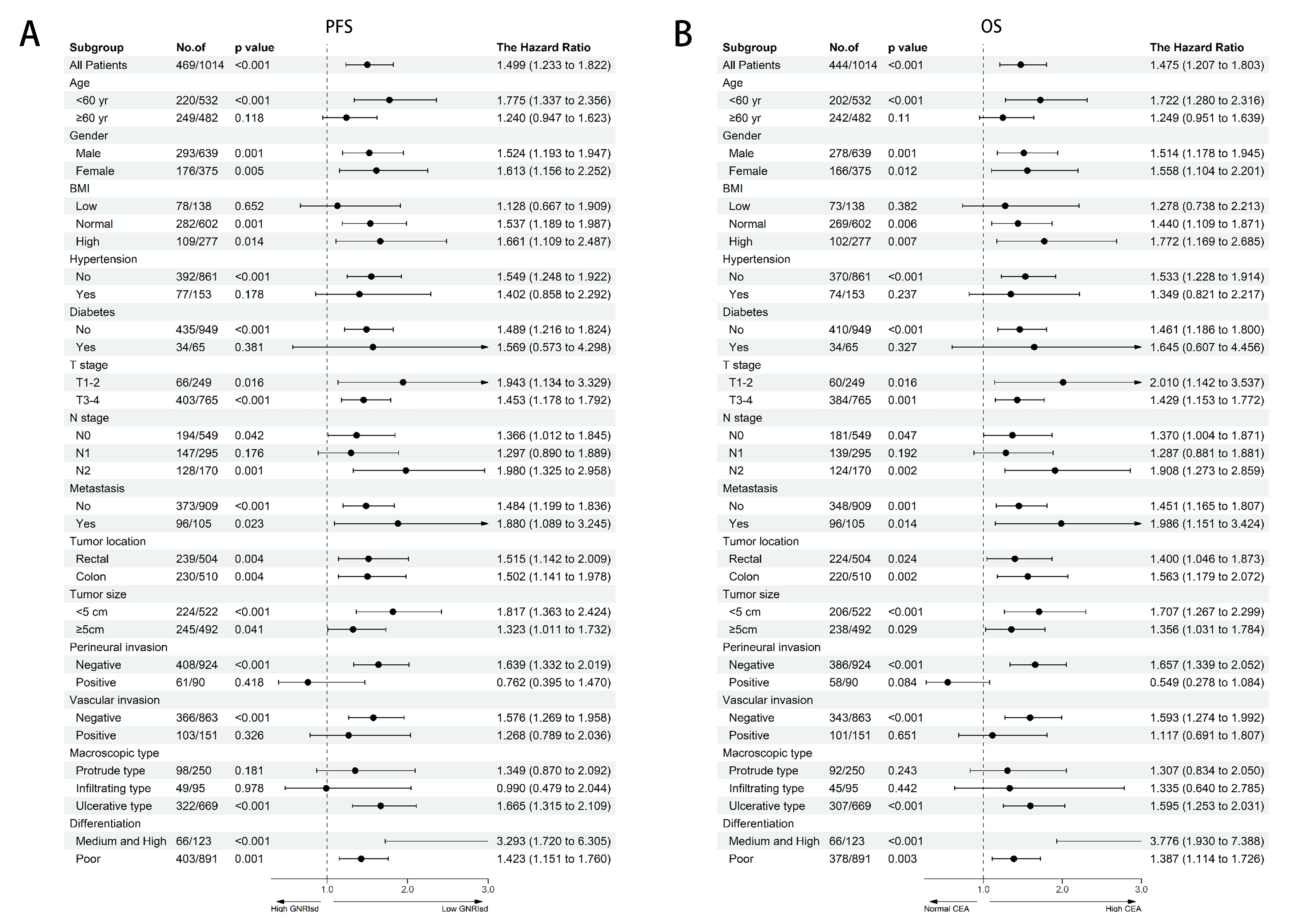


**Figure S8.** Calibration curve of the PFS (A) and OS (B) nomograms.

**
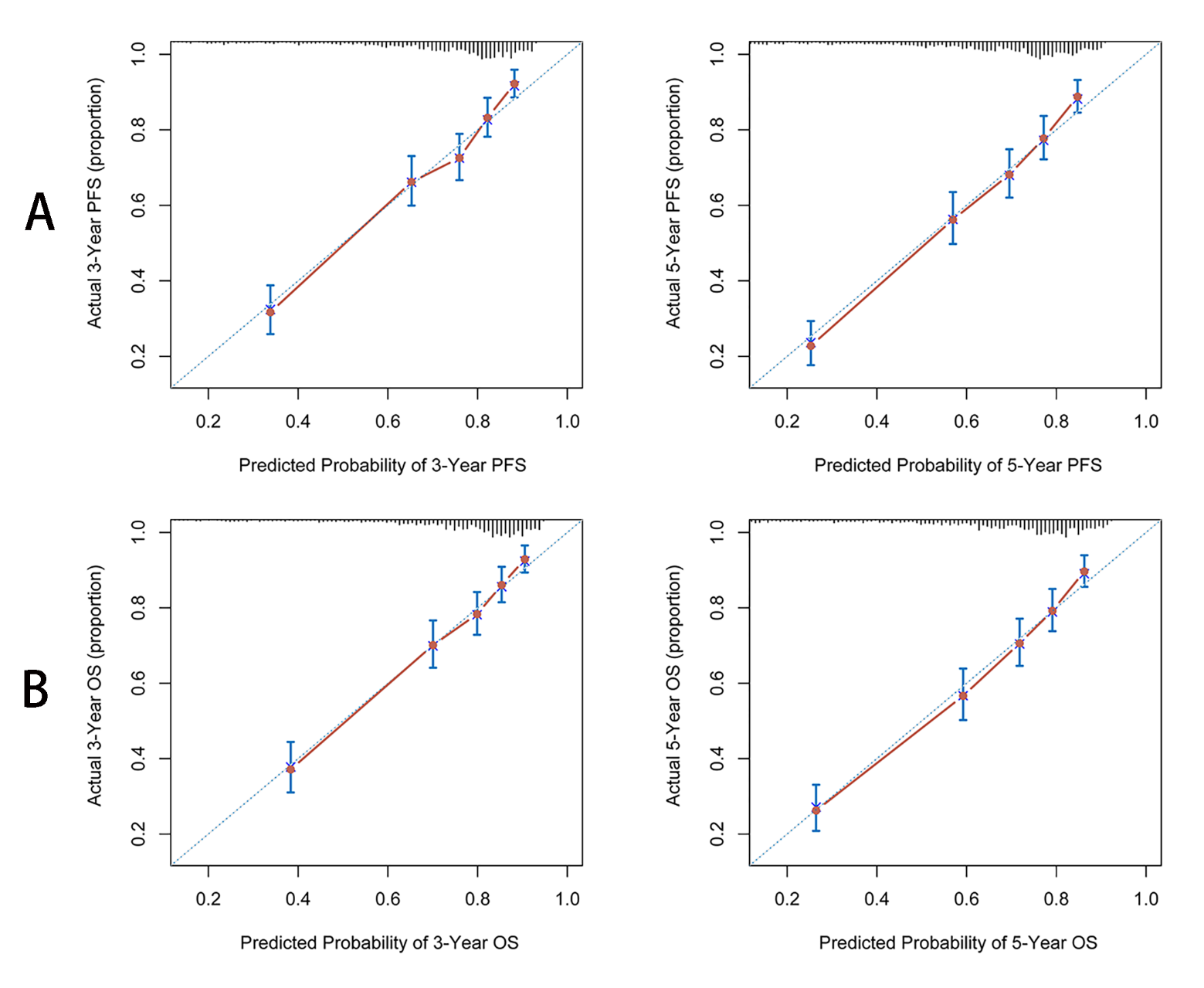
**

**Figure S9.** Comparison of the ability of the novel prognostic nomograms and TNM classification in predicting PFS (A) and OS (B) of CRC patients.

**
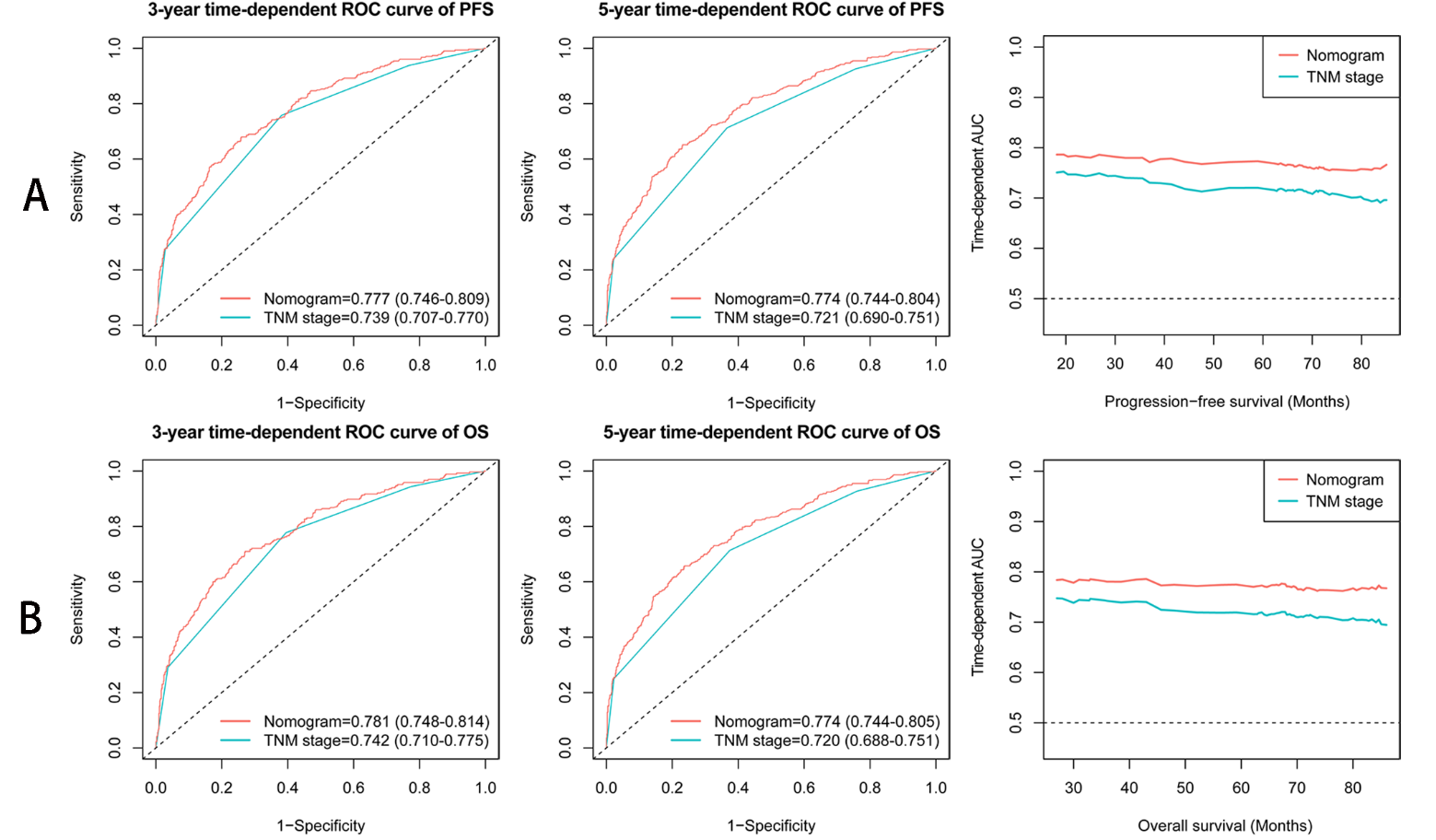
**

**Figure S10.** Time-dependent ROC curve and Kaplan-Meier curve of the PFS (A) and OS (B) nomogram.

**
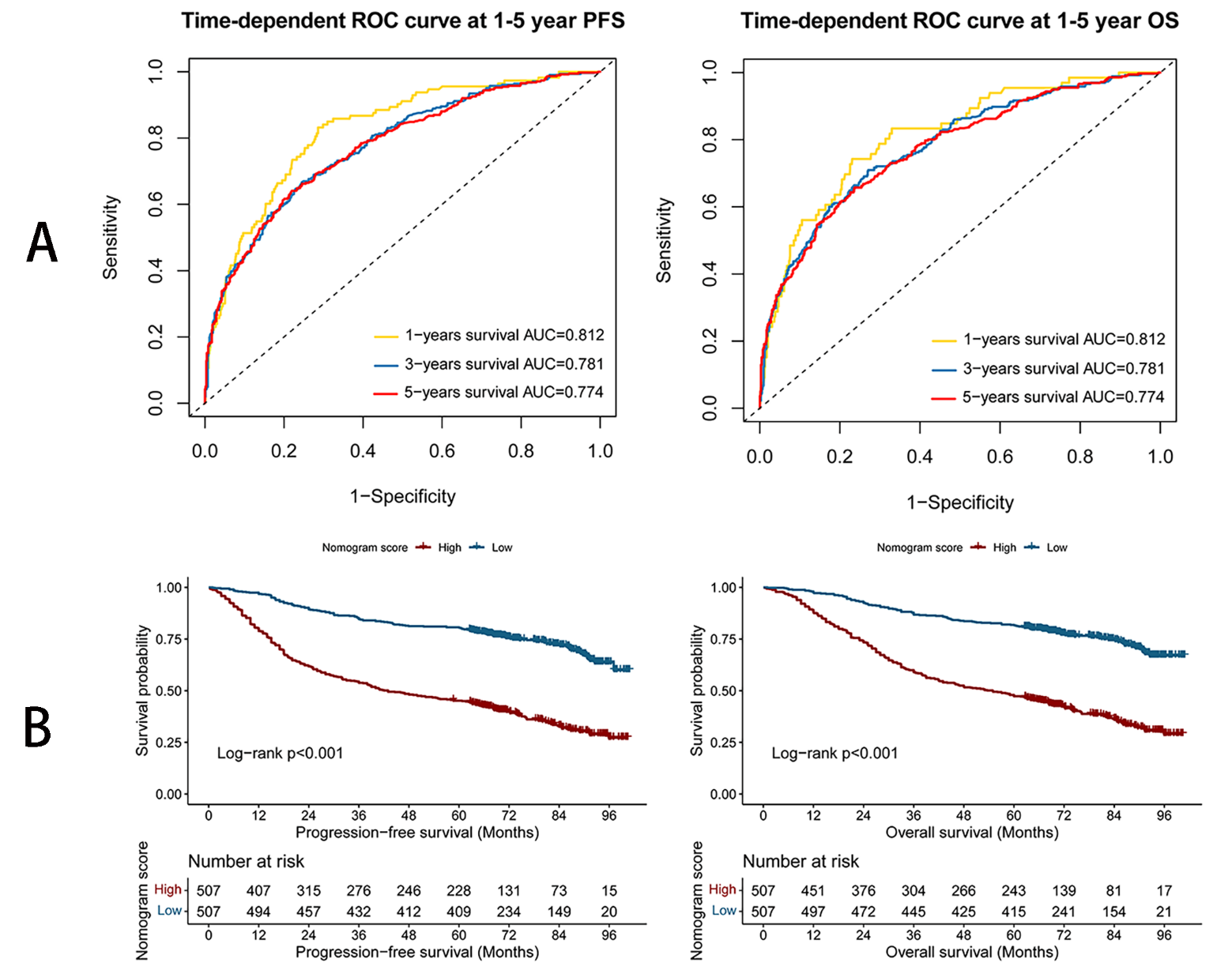
**

**Figure S11.** Randomize internal validation of the novel prognostic nomograms at a ratio of 7:3.

**
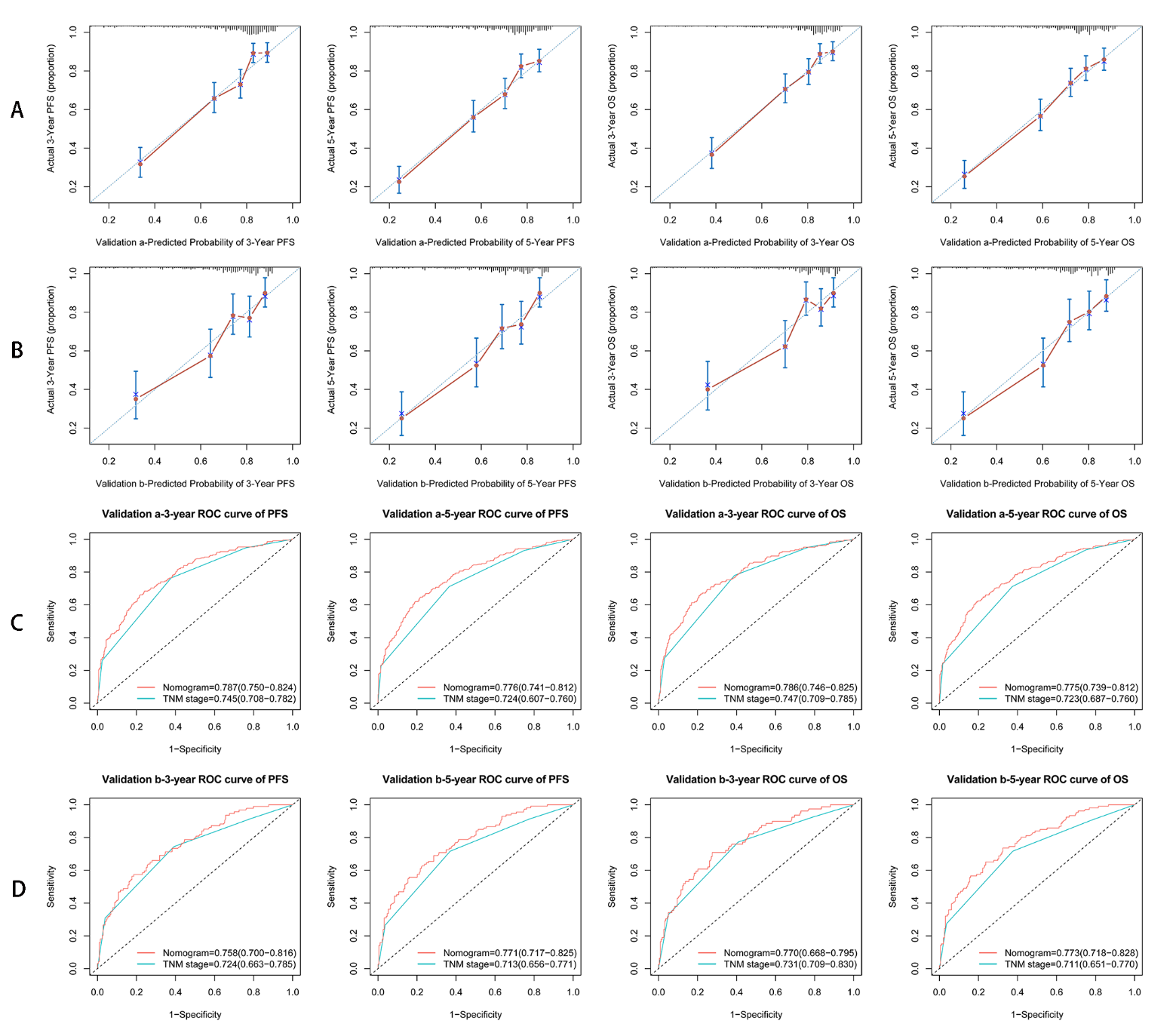
**

**Notes:** A, Calibration curve of validation a; B, Calibration curve of validation b; C, Comparison of mGNRI-TSF score and TNM satge in validation a; D, Comparison of mGNRI-TSF score and TNM satge in validation b.

**Figure S12.** Time-dependent ROC curve and Kaplan-Meier curve of nomograms in validation a and validation b.

| 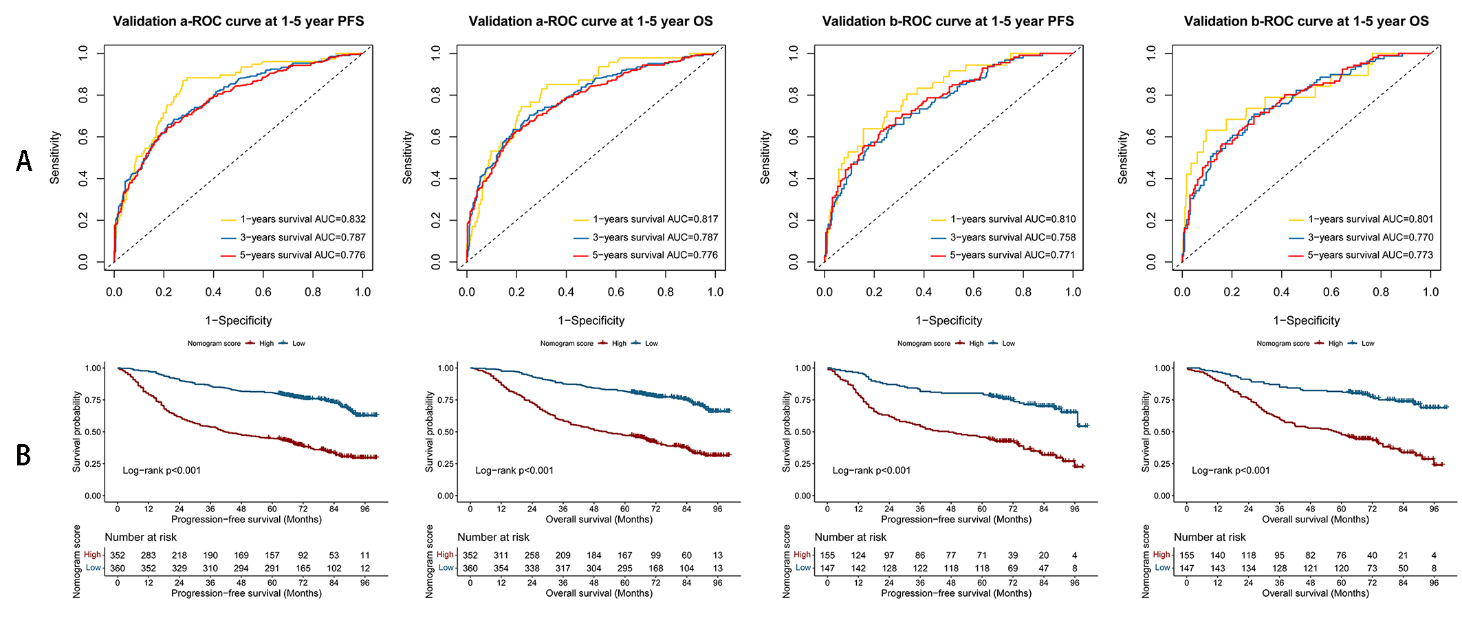 |
| --- |

**Notes:** A, Time-dependent ROC curve of nomogram at 1,3,5 year point; B, Kaplan-Meier curve of nomograms.

**Table S1.** Clinicopathological factors of CRC patients.

| **Characteristic** | Overall | GNRI | | | | CEA | | |
| --- | --- | --- | --- | --- | --- | --- | --- | --- |
|  | n=1014 | High risk, n=243 | Low risk, n=303 | Normal, n=468 | p | Normal, n=586 | High, n=428 | p |
| Gender, male, n (%) | 639 (63.0) | 168 (69.1) | 189 (62.4) | 282 (60.3) | 0.064 | 355 (60.6) | 284 (66.4) | 0.069 |
| Age, years, mean (SD) | 57.33 (13.34) | 60.38 (14.64) | 58.80 (12.91) | 54.80 (12.43) | <0.001 | 56.33 (13.56) | 58.71(12.92) | 0.005 |
| BMI, kg/m2, mean (SD) | 22.12 (3.34) | 20.01 (3.43) | 22.04 (3.03) | 23.26 (2.92) | <0.001 | 22.17 (3.33) | 22.05 (3.36) | 0.594 |
| Hypertension, yes, n (%) | 153 (15.1) | 39 (16.0) | 52 (17.2) | 62 (13.2) | 0.297 | 76 (13.0) | 77 (18.0) | 0.034 |
| Diabetes, yes, n (%) | 65 (6.4) | 17 (7.0) | 23 (7.6) | 25 (5.3) | 0.42 | 32 (5.5) | 33 (7.7) | 0.189 |
| T stage, T3-4, n (%) | 765 (75.4) | 185 (76.1) | 236 (77.9) | 344 (73.5) | 0.37 | 398 (67.9) | 367 (85.7) | <0.001 |
| N stage, n (%) |  |  |  |  | 0.247 |  |  | <0.001 |
| N0 | 549 (54.1) | 143 (58.8) | 166 (54.8) | 240 (51.3) |  | 349 (59.6) | 200 (46.7) |  |
| N1 | 295 (29.1) | 62 (25.5) | 93 (30.7) | 140 (29.9) |  | 157 (26.8) | 138 (32.2) |  |
| N2 | 170 (16.8) | 38 (15.6) | 44 (14.5) | 88 (18.8) |  | 80 (13.7) | 90 (21.0) |  |
| Metastasis, yes, n (%) | 105 (10.4) | 39 (16.0) | 32 (10.6) | 34 (7.3) | 0.001 | 27 (4.6) | 78 (18.2) | <0.001 |
| TNM stage, n (%) |  |  |  |  | 0.004 |  |  | <0.001 |
| Stage I | 184 (18.1) | 49 (20.2) | 49 (16.2) | 86 (18.4) |  | 144 (24.6) | 40 (9.3) |  |
| Stage II | 328 (32.3) | 76 (31.3) | 107 (35.3) | 145 (31.0) |  | 194 (33.1) | 134 (31.3) |  |
| Stage III | 397 (39.2) | 79 (32.5) | 115 (38.0) | 203 (43.4) |  | 221 (37.7) | 176 (41.1) |  |
| Stage IV | 105 (10.4) | 39 (16.0) | 32 (10.6) | 34 (7.3) |  | 27 (4.6) | 78 (18.2) |  |
| Tumor location, rectal, n (%) | 504 (49.7) | 93 (38.3) | 149 (49.2) | 262 (56.0) | <0.001 | 304 (51.9) | 200 (46.7) | 0.120 |
| Tumor size (median (IQR)) | 4.50 (2.50) | 5.50 (2.5) | 5.00 (2.00) | 4.00 (2.00) | <0.001 | 4.50 (2.50) | 5.00 (2.00) | <0.001 |
| Perineural invasion, positive, n (%) | 90 (8.9) | 22 (9.1) | 23 (7.6) | 45 (9.6) | 0.624 | 45 (7.7) | 45 (10.5) | 0.145 |
| Vascular invasion, positive, n (%) | 151 (14.9) | 29 (11.9) | 49 (16.2) | 73 (15.6) | 0.324 | 77 (13.1) | 74 (17.3) | 0.081 |
| Macroscopic type, n (%) |  |  |  |  | 0.035 |  |  | 0.012 |
| Protrude type | 250 (24.7) | 75 (30.9) | 72 (23.8) | 103 (22.0) |  | 163 (27.8) | 87 (20.3) |  |
| Infiltrating type | 95 (9.4) | 25 (10.3) | 33 (10.9) | 37 (7.9) |  | 58 (9.9) | 37 (8.6) |  |
| Ulcerative type | 669 (66.0) | 143 (58.8) | 198 (65.3) | 328 (70.1) |  | 365 (62.3) | 304 (71.0) |  |
| Differentiation, poor, n (%) | 891 (87.9) | 209 (86.0) | 274 (90.4) | 408 (87.2) | 0.239 | 513 (87.5) | 378 (88.3) | 0.783 |
| White blood cell (median (IQR)) | 6.70 (2.57) | 7.01 (3.08) | 6.60 (2.41) | 6.60 (2.41) | 0.121 | 6.53 (2.57) | 6.80 (2.47) | 0.028 |
| Hemoglobin (median (IQR)) | 118.85 (28.95) | 105.00 (34.35) | 116.60 (23.50) | 125.65 (23.87) | <0.001 | 119.95 (27.2) | 116.75 (30.45) | 0.023 |
| Neutrophil (median (IQR)) | 3.87 (2.02) | 4.41 (2.76) | 3.84 (1.88) | 3.79 (1.76) | 0.001 | 3.80 (1.91) | 4.04 (2.11) | 0.001 |
| Lymphocyte (median (IQR)) | 1.76 (0.80) | 1.54 (0.75) | 1.73 (0.66) | 1.91 (0.84) | <0.001 | 1.80 (0.80) | 1.70 (0.77) | 0.007 |
| Albumin (median (IQR)) | 38.10 (5.30) | 33.00 (4.45) | 36.80 (1.85) | 40.80 (3.00) | <0.001 | 38.40 (4.90) | 37.75 (5.70) | 0.006 |
| Length of stay (median (IQR)) | 18.00 (6.00) | 20.00 (7.00) | 19.00 (6.00) | 18.00 (6.00) | <0.001 | 18.00 (6.00) | 19.00 (6.00) | 0.323 |
| Recurrence and metastasis, yes, n (%) | 297 (29.3) | 88 (36.2) | 93 (30.7) | 116 (24.8) | 0.005 | 133 (22.7) | 164 (38.3) | <0.001 |
| Death, yes, n (%) | 444 (43.8) | 140 (57.6) | 140 (46.2) | 164 (35.0) | <0.001 | 204 (34.8) | 240 (56.1) | <0.001 |

**Table S2.** Comparative analysis of the discrimination of GNRI, CEA, GNRI-CEA score for overall survival in patients with cancer.

| Discrimination Ability | C-statistic | | cNRI | | IDI | |
| --- | --- | --- | --- | --- | --- | --- |
|  | Difference | p value | Difference | p value | Difference | p value |
| mGNRI-TSF score | Ref |  | Ref |  | Ref |  |
| mGNRI | -0.043(-0.057,-0.029) | <0.001 | -0.224(-0.282, -0.161) | <0.001 | -0.030(-0.045, -0.017) | <0.001 |
| CEA | -0.013(-0.037, 0.011) | 0.288 | -0.037(-0.105, 0.028) | 0.236 | -0.003(-0.027, 0.021) | 0.797 |
| Model performance after the addition of other indexes to the TNM stage for predicting all-cause mortality | | | | | | |
| Model | C-statistic | P value | cNRI | p value | IDI | p value |
| TNM stage | 0.681(0.656,0.705) | <0.001 | Ref |  | Ref |  |
| TNM stage +GNRI-CEA score | 0.704(0.679,0.729) | <0.001 | 0.175(0.082,0.227) | <0.001 | 0.026(0.010,0.045) | <0.001 |
| TNM stage + GNRI | 0.696(0.671,0.721) | <0.001 | 0.114(0.047,0.178) | 0.010 | 0.014(0.003,0.030) | 0.010 |
| TNM stage + CEA | 0.698(0.673,0.723) | <0.001 | 0.224(0.162,0.290) | <0.001 | 0.018(0.005,0.035) | 0.002 |

Table note: cNRI, continuous net reclassification improvement; IDI, integrated discrimination improvement.

**Table S3.** Comparative analysis of the discrimination of GNRI, CEA, GNRI-CEA score for progression-free survival in patients with cancer.

| Discrimination Ability | C-statistic | | cNRI | | IDI | |
| --- | --- | --- | --- | --- | --- | --- |
|  | Difference | p value | Difference | p value | Difference | p value |
| mGNRI-TSF score | Ref |  | Ref |  | Ref |  |
| mGNRI | -0.044(-0.058,-0.031) | <0.001 | -0.225(-0.282, -0.162) | <0.001 | -0.030(-0.044, -0.018) | <0.001 |
| CEA | -0.011(-0.033, 0.013) | 0.366 | -0.043(-0.103, 0.021) | 0.186 | -0.004(-0.028, 0.021) | 0.729 |
| Model performance after the addition of other indexes to the TNM stage for predicting all-cause mortality | | | | | | |
| Model | C-statistic | P value | cNRI | p value | IDI | p value |
| TNM stage | 0.679(0.656,0.702) | <0.001 | Ref |  | Ref |  |
| TNM stage +GNRI-CEA score | 0.702(0.678,0.725) | <0.001 | 0.190(0.099,0.251) | <0.001 | 0.028(0.012,0.049) | <0.001 |
| TNM stage + GNRI | 0.693(0.669,0.717) | <0.001 | 0.124(0.054,0.187) | 0.006 | 0.016(0.003,0.032) | 0.004 |
| TNM stage + CEA | 0.697(0.673,0.720) | <0.001 | 0.225(0.162,0.288) | <0.001 | 0.019(0.006,0.035) | <0.001 |

Table note: cNRI, continuous net reclassification improvement; IDI, integrated discrimination improvement.

**Table S4.** Cox regression analysis of characteristics associated with progression-free survival in CRC patients.

| Characteristics | Univariate analysis | | Multivariate analysis | |
| --- | --- | --- | --- | --- |
|  | HR, 95% CI | p value | HR.CI95 | p value |
| Gender (male) | 0.988 (0.819-1.191) | 0.895 |  |  |
| Age | 1.007 (1-1.014) | 0.042 | 1.010 (1.002 - 1.017) | 0.008 |
| BMI | 0.952 (0.926-0.979) | 0.001 | 0.998 (0.969 - 1.028) | 0.888 |
| T stage (T3-4) | 2.461 (1.897-3.194) | <0.001 | 1.590 (1.208 - 2.093) | 0.001 |
| N stage |  | <0.001 |  | <0.001 |
| N0 | ref |  | ref |  |
| N1 | 1.649 (1.331-2.044) | <0.001 | 1.424 (1.14 - 1.779) | 0.002 |
| N2 | 3.636 (2.902-4.554) | <0.001 | 2.737 (2.144 - 3.494) | <0.001 |
| Metastasis (Yes) | 5.319 (4.223-6.699) | <0.001 | 3.361 (2.615 - 4.319) | <0.001 |
| Perineural invasion (Positive) | 1.808 (1.381-2.366) | <0.001 | 1.121 (0.831 - 1.512) | 0.454 |
| Vascular invasion (Positive) | 1.986 (1.595-2.472) | <0.001 | 1.419 (1.106 - 1.821) | 0.006 |
| Differentiation (Poor) | 0.709 (0.546-0.92) | 0.01 | 0.878 (0.667 - 1.154) | 0.35 |
| Tumor size (≥5 cm) | 1.239 (1.033-1.485) | 0.021 | 0.896 (0.737 - 1.088) | 0.266 |
| Surgical approach (Laparoscope) | 0.652 (0.543-0.782) | <0.001 | 0.868 (0.714 - 1.055) | 0.155 |
| GNRI-CEA score |  | <0.001 |  | <0.001 |
| Normal | ref |  | ref |  |
| Mild | 1.529 (1.176-1.988) | 0.002 | 1.34 (1.023 - 1.756) | 0.034 |
| Moderate | 2.207 (1.697-2.87) | <0.001 | 1.949 (1.458 - 2.605) | <0.001 |
| Severe | 3.093 (2.288-4.181) | <0.001 | 2.257 (1.6 - 3.183) | <0.001 |

**Table S5.** Cox regression analysis of characteristics associated with overall survival in CRC patients.

| Characteristics | Univariate analysis | | Multivariate analysis | |
| --- | --- | --- | --- | --- |
|  | HR, 95% CI | p value | HR.CI95 | p value |
| Gender (male) | 0.989 (0.816-1.199) | 0.910 |  |  |
| Age | 1.010 (1.002-1.017) | 0.010 | 1.012 (1.005 - 1.019) | 0.001 |
| BMI | 0.953 (0.926-0.981) | 0.001 | 1.000 (0.970 - 1.031) | 0.996 |
| T stage (T3-4) | 2.539 (1.934-3.334) | <0.001 | 1.626 (1.221 - 2.165) | 0.001 |
| N stage |  | <0.001 |  | <0.001 |
| N0 | ref |  | ref |  |
| N1 | 1.628 (1.305-2.031) | <0.001 | 1.395 (1.109 - 1.754) | 0.004 |
| N2 | 3.665 (2.91-4.615) | <0.001 | 2.708 (2.109 - 3.476) | <0.001 |
| Metastasis (Yes) | 5.765 (4.57-7.274) | <0.001 | 3.579 (2.779 - 4.608) | <0.001 |
| Perineural invasion (Positive) | 1.791 (1.359-2.361) | <0.001 | 1.078 (0.794 - 1.465) | 0.629 |
| Vascular invasion (Positive) | 2.046 (1.638-2.555) | <0.001 | 1.468 (1.14 - 1.891) | 0.003 |
| Differentiation (Poor) | 0.656 (0.505-0.852) | 0.002 | 0.802 (0.609 - 1.057) | 0.117 |
| Tumor size (≥5 cm) | 1.341 (1.112-1.616) | 0.002 | 0.97 (0.794 - 1.184) | 0.763 |
| Surgical approach (Laparoscope) | 0.618 (0.513-0.745) | <0.001 | 0.845 (0.691 - 1.033) | 0.101 |
| GNRI-CEA score |  | <0.001 |  | <0.001 |
| Normal | ref |  | ref |  |
| Mild | 1.531 (1.167-2.007) | 0.002 | 1.302 (0.984 - 1.722) | 0.064 |
| Moderate | 2.2 (1.678-2.886) | <0.001 | 1.883 (1.398 - 2.536) | <0.001 |
| Severe | 3.258 (2.394-4.436) | <0.001 | 2.242 (1.579 - 3.185) | <0.001 |
